# Miller Fisher Syndrome and Boomerang Sign: A Rare Presentation of Typhoid Fever

**DOI:** 10.7759/cureus.15386

**Published:** 2021-06-02

**Authors:** Boby Varkey Maramattom, Balram Rathish, Hanna A Meleth

**Affiliations:** 1 Neurology, Aster Medcity, Kochi, IND; 2 Infectious Diseases, Aster Medcity, Kochi, IND

**Keywords:** guillain-barré syndrome, typhoid, boomerang sign, miller fisher syndrome, guillain-barré

## Abstract

Miller Fischer syndrome (MFS) is a variant of Guillain-Barré syndrome which is characterized by a triad of ataxia, ophthalmoplegia, and areflexia. It is uncommonly associated with systemic illnesses. We present the case of a young boy who came back after a trip to New Delhi in India, who developed MFS. MRI showed a callosal splenial hyperintensity which is called the boomerang sign. The evaluation revealed typhoid fever. This is probably the first such report of MFS associated with this infection.

## Introduction

Miller Fischer syndrome (MFS) is a variant of Guillain-Barré syndrome (GBS) characterized by ataxia, ophthalmoplegia, and areflexia [1]. It has a better prognosis than classic GBS and most patients recover uneventfully [1]. GBS has been reported in association with typhoid fever as well as Bickerstaff brainstem encephalitis in association with *Salmonella paratyphi* infections; however, MFS has not been reported yet in association with typhoid fever in the published literature [2,3]. We present an unusual association of MFS with typhoid fever.

## Case presentation

A 16-year-old boy, a national-level swimmer, presented with a headache for three days, high-grade fever, and loose stools. He had a history of travel to New Delhi two weeks prior to the onset of symptoms. On day two, he developed horizontal diplopia, with slurring of speech and an unsteady gait, with a tendency to fall to the left. He was then afebrile for two days and brought for admission on day five of illness. Examination revealed relative bradycardia, mild dysarthria, upbeat nystagmus and gaze-evoked horizontal nystagmus, truncal titubation, limb ataxia, and generalized areflexia. He had palpable mild hepatosplenomegaly. With his triad of ataxia, areflexia, and ophthalmoplegia, a diagnosis of MFS variant of GBS was considered. Liver function tests showed mildly elevated transaminases. Rapid card tests for scrub typhus, malaria, dengue, and leptospira were negative. Nerve conduction studies showed features of a demyelinating sensorimotor polyneuropathy involving both the upper and lower limbs. Visual evoked potential studies showed mild prolongation of P100 latencies from the left eye.

**Figure 1:**
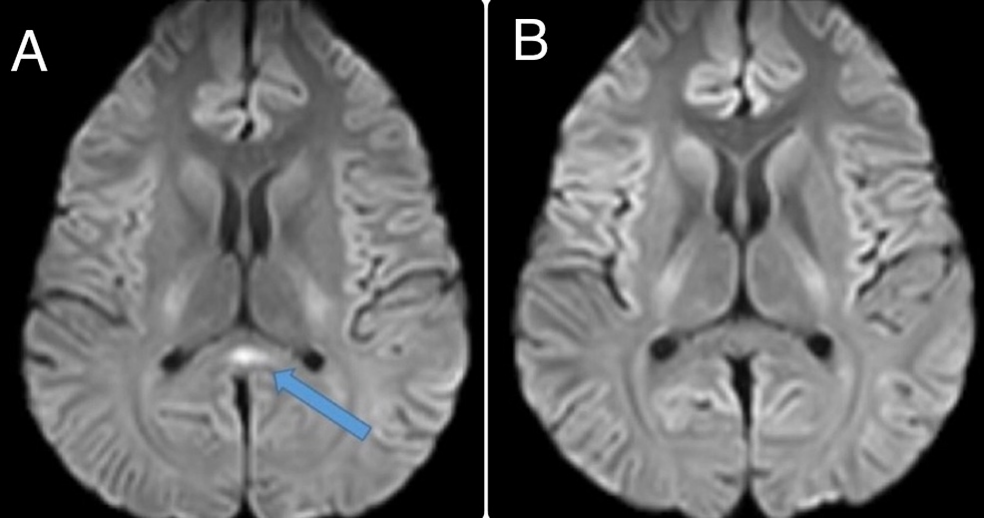
MRI brain showing the boomerang sign and its resolution after treatment. (A) First MRI showing a small splenial diffusion-weighted MRI hyperintensity (blue arrow). (B) Repeat MRI done after one week shows resolution of splenial hyperintensity.

A diagnosis of MFS was made. When fever recurred, an underlying infection was also considered. The differential diagnosis of a secondary MFS included dengue, West Nile virus, cytomegalovirus, Epstein-Barr virus, influenza, HIV, Zika virus, and *Campylobacter jejuni*. MRI brain on day five of illness showed a small splenial hyperintensity on diffusion-weighted sequences called the boomerang sign (Figure 1). In view of the splenial boomerang sign, other infections such as *Salmonella*, varicella-zoster virus, and tuberculous meningitis were also considered. Whole spine MRI was normal. From day six onwards, he started spiking high fevers. A cerebrospinal fluid (CSF) study showed a total of three cells with protein of 42.8 mg% and no albuminocytological dissociation. A rapid card test for *Salmonella *immunoglobulin M (IgM) was positive and blood cultures grew *Salmonella typhi* by day six. CSF tests for West Nile virus, Japanese encephalitis, and Epstein-Barr virus were negative. An anti-GQ1b IgG antibody test was negative.

Intravenous immunoglobulin (IVIg) was started at a dose of 2 g/kg over five days on day five. On day six, after blood cultures grew *Salmonella*, ceftriaxone was added. A good prognosis was expected as his MFS symptoms were mild and thought to be reversible. Furthermore, typhoid fever also seemed to be responding to ceftriaxone with a general feeling of wellness. A final diagnosis of parainfectious MFS with typhoid was made. He continued to be febrile for six more days until day 11 although his diarrhea subsided by day eight, and diplopia and dysarthria improved by day 10. A repeat MRI on day 11 showed resolution of the spenial hyperintensity (Figure 1). He was discharged on day 14. At follow-up 10 days later, he was asymptomatic.

## Discussion

Typhoid fever is associated with a wide variety of neurological manifestations including encephalopathy, spastic paralysis, seizures, meningitis, Parkinsonian syndromes, sensorimotor neuropathy, cerebellar involvement, and schizophrenic psychosis [4,5]. However, GBS associated with typhoid is very rare and the MFS variant of GBS has not yet been reported in typhoid or salmonellosis. MFS has been reported as a parainfectious manifestation of dengue, *Mycoplasma pneumoniae*, and Zika virus infection [6-8].

The clinical hallmark of MFS is a triad of acute ophthalmoplegia, areflexia, and ataxia in the setting of a preceding bacterial or viral illness. Both MFS and GBS are thought to result from an aberrant acute autoimmune response to a preceding infection. A cross-reaction between peripheral nerve antigens and microbial/viral components through molecular mimicry probably drives the inflammatory process of this illness [5,6]. This is probably the first case of MFS occurring as a parainfectious complication of typhoid fever.

Our patient also demonstrated the splenial boomerang sign on MRI. This refers to a cytotoxic lesion of the corpus callosum and is known by a variety of names including transient lesions of the splenium of the corpus callosum, mild encephalitis/encephalopathy with a reversible isolated corpus callosum lesion, and reversible splenial lesion syndrome. This is often transient and associated with a wide variety of etiologies, as summarized in Table 1 [9-11].

**Table 1:** Causes of transient splenial hyperintensity.

Category	Cause
Infections	Cytomegalovirus
	West Nile virus
	Dengue
	Salmonella
	Malaria
	Rotavirus
	Tuberculous meningitis
Demyelinating disorders	Acute disseminated encephalomyelitis
	Systemic lupus erythematosus
Metabolic	Hypoglycemia
	Hyponatremia
	Hypernatremia
	Hyperammonemia
	Extrapontine myelinolysis
	Renal failure
Vascular	Cerebrovascular disease
	Post cardiac arrest
	Hypertensive encephalopathy
	Pre-eclampsia
	Posterior reversible encephalopathy syndrome
	Migraine with aura
	Subarachnoid hemorrhage
Miscellaneous	Malnutrition: vitamin B12 deficiency
	Drug toxicity: cyclosporine, fluorouracil, metronidazole
	High-altitude cerebral edema
	Traumatic: axonal injury
	Hemolytic uremic syndrome
	Leptomeningeal metastases
Epilepsy	Seizures
	Abrupt drug withdrawal
	Antiepileptic drug overdose

## Conclusions

In conclusion, typhoid fever can cause a parainfectious MFS. Salmonellosis is also known to cause a transient splenial boomerang sign on MRI. Both typhoid and MFS can be expected to improve with treatment. This is probably the first report of MFS as a parainfectious complication of typhoid fever. In similar cases with MFS or GBS with continuing fever, it is important to search for the underlying cause and treat it appropriately.
